# Novel hybrid organic/inorganic 2D quasiperiodic PC: from diffraction pattern to vertical light extraction

**DOI:** 10.1186/1556-276X-6-371

**Published:** 2011-05-04

**Authors:** Lucia Petti, Massimo Rippa, Jun Zhou, Liberato Manna, Marco Zanella, Pasquale Mormile

**Affiliations:** 1 Institute of Cybernetics "E. Caianiello" of CNR, Via Campi Flegrei 34, 80072 Pozzuoli, Italy; 2 Institute of Photonics, Faculty of Science, Ningbo University, Ningbo, Zhejiang 315211, China; 3 Fondazione Istituto Italiano di Tecnologia, via Morego 30, 16163, Genova, Italy

## Abstract

Recently, important efforts have been dedicated to the realization of a fascinating class of new photonic materials or metamaterials, known as photonic quasicrystals (PQCs), in which the lack of the translational symmetry is compensated by rotational symmetries not achievable by the conventional periodic crystals. As ever, more advanced functionality is demanded and one strategy is the introduction of non-linear and/or active functionality in photonic materials. In this view, core/shell nanorods (NRs) are a promising active material for light-emitting applications. In this article a two-dimensional (2D) hybrid a 2D octagonal PQC which consists of air rods in an organic/inorganic nanocomposite is proposed and experimentally demonstrated. The nanocomposite was prepared by incorporating CdSe/CdS core/shell NRs into a polymer matrix. The PQC was realized by electron beam lithography (EBL) technique. Scanning electron microscopy, far field diffraction and spectra measurements are used to characterize the experimental structure. The vertical extraction of the light, by the coupling of the modes guided by the PQC slab to the free radiation via Bragg scattering, consists of a narrow red emissions band at 690 nm with a full width at half-maximum (FWHM) of 21.5 nm. The original characteristics of hybrid materials based on polymers and colloidal NRs, able to combine the unique optical properties of the inorganic moiety with the processability of the host matrix, are extremely appealing in view of their technological impact on the development of new high performing optical devices such as organic light-emitting diodes, ultra-low threshold lasers, and non-linear devices.

**PACS: **81.07.Pr Organic-inorganic hybrid nanostructures, 81.16.-c Methods of nanofabrication and processing, 42.70.Qs Photonic band-gap materials.

## Background

Quasiperiodic crystals are a new class of materials that exhibit long-range aperiodic translational order and high rotational symmetries [1,2]. Unlike periodically arranged photonic crystals (or photonic band-gaps), photonic quasicrystals (PQCs) possess unique light localization and transport properties related to their complex, multi-fractal energy spectra [3-14].

PQCs possess photonic band-gaps (PBGs) with very interesting properties of light transmission [15,16], wave guiding, and localization [17-19] exploited in an enormous variety of electro-optical and photonic applications. The existence of high rotational symmetries and many not equivalent defective states [20] not achievable by conventional periodic crystals opens the possibility to realize versatile, robust PBG devices even for low dielectric contrast materials like polymeric ones. Different from random structures, PQCs are defined by the iteration of simple mathematical rules, rooted in symbolic dynamics and prime number theory, which possess very rich spectral features [5]. The structural complexity of PQCs is measured by their spatial Fourier spectra, which are discrete (singular) for quasiperiodic systems, singular-continuous, or absolutely continuous for pseudorandom structures of increasing complexity. Two-dimensional (2D) quasiperiodic lattices possessing high rotational symmetries have been largely studied in literature [21-26]. The experimental realization of 2D PQCs is a hard fabrication challenge. Two-beam and multiple-beam holographic lithography have been largely employed to realize periodic [27] and quasiperiodic [28] crystals at the mesoscale. Moreover, novel PBG aperiodic structures defined by recursive substitutional sequences like one-dimensional (1D) and 2D Thue-Morse patterns cannot be realized even in principle by multiple-beam interference. Experimental realization of photonic structures exhibiting large area 2D Thue-Morse arrangement has been recently reported by the authors for the first time to our knowledge [29].

Advances in 2D photonic structures are expected in the introduction of non-linear and/or active functionality into a 2D PQC. 1D semiconductor nanostructures are likewise promising materials both in fundamental research and in practical applications [30-33]. As an example, it has been shown that various types of semiconductor nanorods (NRs) have unique optical properties that make them appealing for applications in solar cells, light-emitting diodes, lasers, and cell labeling [33-36]. CdSe/CdS rods present the appealing characteristics of strong and tunable light emission from green to red, are highly fluorescent and show linearly polarized emission [37].

These characteristics open the way to a new class of hybrid devices based on polymers and colloidal NRs in which the unique optical properties of the inorganic moiety are combined with the processability of the host matrix to develop new high performing optical devices such as organic light-emitting diodes, ultra-low threshold lasers, and non-linear devices. One of the challenges of these applications is the incorporation of inorganic nanoparticles into organic polymer matrices, since this is usually accompanied by phase separation, aggregation of nanoparticles, loss of transparency, and luminescence quenching due to exciton energy transfer [38,39].

In this paper, for the first time to our knowledge, a 2D hybrid eightfold symmetric aperiodically ordered PQC which consists of air rods in a nanocomposite prepared by incorporating CdSe/CdS core/shell nanorods (NRs) in a polymer is proposed and experimentally demonstrated. The semiconductor nanocrystal based 2D PQC was fabricated by directly patterning and releasing a thin film of a functional material composed by NRs dispersed in a positive electronic resist.

Scanning electron microscopy (SEM) and optical measurements are used to characterize the structures. The vertical extraction of the light, by the coupling of the modes guided by the PQCs slab to the free radiation via Bragg scattering, consists of a narrow red emissions band at 690 nm with a FWHM of 21.5 nm.

## Methods

### Synthesis and fabrication

**Figure 1:**
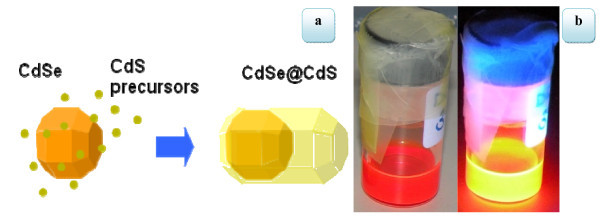
**Light emission properties of the colloidal solution**. **(a) **Sketch of the seeded growth approach for the preparation of CdSe/CdS nanorods and **(b) **pictures of nanorods in toluene under visible light and UV showing photoluminescence.

The dot/rod core/shell CdSe/CdS nanocrystals were synthesized by a "seeded growth" approach [40] which yields highly uniform nanocrystals in terms of distribution of rod lengths and diameters that additionally emit wavelength tunable, linearly polarized light with high quantum efficiency. In particular, the synthetic procedure relied on the rapid co-injection of preformed spherical CdSe nanocrystal seeds and elemental sulphur dissolved in trioctylphosphine, in a reaction flask that contains a solution of cadmium oxide in a mixture of trioctylphosphine oxide, hexylphosphonic acid, and octadecylphosphonic acid at high temperature (350°C) [40]. These NRs are dispersed in a solution of polymethylmethacrylate (PMMA) and toluene. In Figure 1a,b we report a sketch showing the seeded growth approach for the preparation of dot/rod core/shell CdSe/CdS nanocrystals and a photograph of the solution under visible light and UV light.

**Figure 2:**
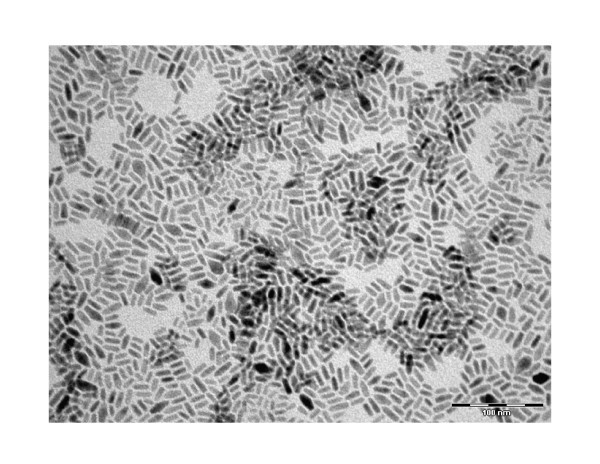
**TEM analysis of the colloidal solution**. A transmission electron micrograph of the stock solution of CdSe/CdS nanorods dissolved in toluene, deposited on copper grid coated with a thin amorphous carbon film, after solvent evaporation. The average rod diameter and length, as determined by TEM were 4 and 20 nm (aspect ratio 5), respectively.

A morphological analysis of the stock solution of CdSe/CdS NRs dissolved in toluene was carried out by means of transmission electron microscopy (TEM) and is reported in Figure 2.

We firstly fabricated CdSe/CdS NRs-PMMA polymer composite film by combining the CdSe/CdS NRs with PMMA of high optical transparency in the visible region and spin-coating the composite solution. Since PMMA is transparent in the visible spectral range and it is an electron-sensitive material, it was chosen as the embedding matrix for NRs. Clear nanocomposite films without bubbles and smooth surfaces with sizes up to 1.5 × 2.5 × 0.0000600 cm^3 ^on an indium tin oxide (ITO) coated glass were obtained.

Our experimental structure was fabricated by using a high-resolution electron beam lithography (EBL) technique. The EBL facility employed consisted of a Raith 150 system. Such system enables the writing of patterns of arbitrary geometries with a spatial resolution up to 10 nm. The samples were obtained by exposing a layer of our mixture of CdSe/CdS NRs doped PMMA deposited on an ITO coated glass. The e-beam is locally focused on the sample to expose selected regions of material homogenously along the depth of the substrate according to the calculated desired pattern. Exposed areas were dissolved away leaving a 2D eightfold octagonal quasiperiodic structure made of air-filled cylinders, located at the vertices of the octagonal lattice, lying in the polymer matrix.

The resulting 2D QC is made of air rods embedded into a hybrid organic/inorganic matrix of CdSe/CdS NRs doped PMMA. The size of the patterned area is about 650 μm^2^. The air rods arrays were fabricated using EBL on quartz substrates with a 15 nm layer ITO for conduction. A 600-nm-thick layer of colloidal doped PMMA was spin-coated on top of the cleaned substrate. Subsequently, the nanopatterning was defined using the Raith 150 system with current and area dosage of 27.8 pA at 20 keV. The resist was developed in a 1:3 solution of methyl isobutyl ketone (MIBK) and isopropanol (IPA).

**Figure 3:**
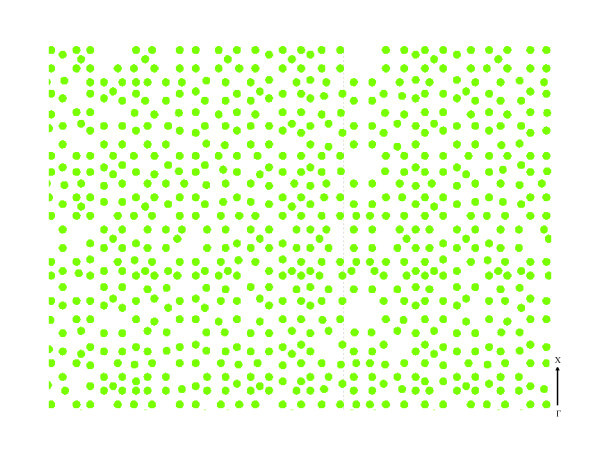
**Pattern design**. Schematic figure showing the octagonal quasiperiodic arrangement of cylinders.

Recently, the importance of tiling geometry in the arrangement of the octagonal quasicrystal was discussed. The fabricated octagonal structure was determined by simulating the quasiperiodic transverse irradiance distribution given by 8-beam interference process and was theoretically analyzed through finite difference time domain (FDTD) simulations of the transmittance spectra [41]. The octagonal tiling gives rise to a wide photonic band-gap for low refractive index difference as low as 0.5 with an attenuation of the transmittance signal up to 50 dB [42]. Therefore, optoelectronics and photonics devices based on such octagonal photonic quasicrystal promise to be realized even in soft materials like polymer. The arrangement of the cylinders is shown schematically in Figure 3.

**Figure 4:**
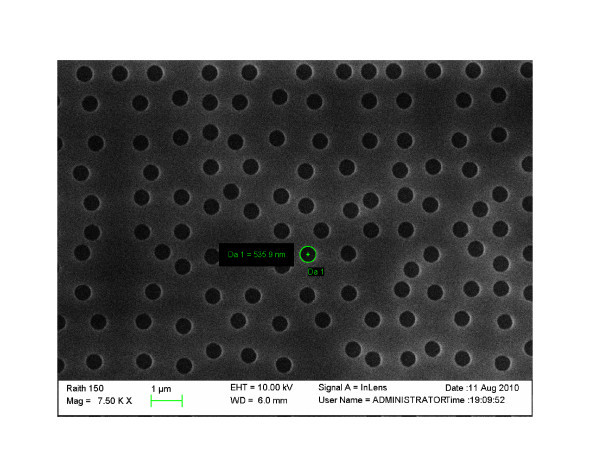
**SEM characterization of the realized hybrid nanostructure**. Top view of SEM image of the 2D PC pattern SEM image characterization of the hybrid eightfold rotational symmetry photonic quasicrystal realized in the colloidal doped PMMA with EBL with a hole diameter *d *= 535 nm.

A SEM image of the eightfold rotational symmetry PQC realized in the hybrid nanocomposite film with a hole diameter of 535 nm is reported in Figure 4.

## Results and discussion

### Diffraction pattern

**Figure 5:**
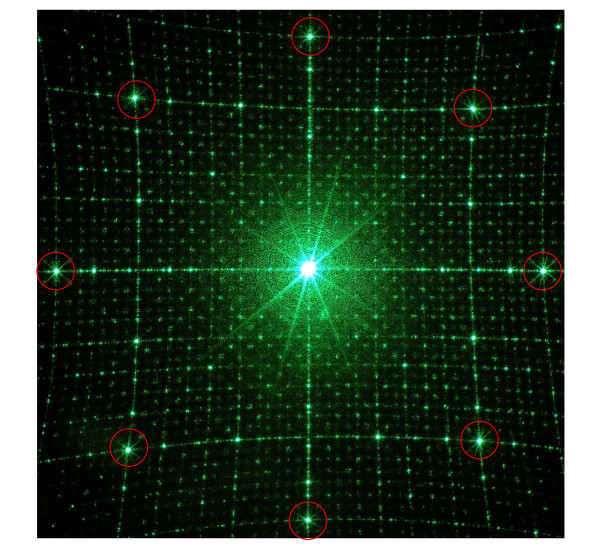
**Far field diffraction characterization of the realized hybrid nanostructure**. Far field diffraction pattern of the 2D octagonal quasicrystal. The red circles indicate the most pronounced peaks on the experimental diffraction pattern.

The experimental octagonal quasicrystal realized has been characterized in the direct space through SEM metrological measurements, whereas in the reciprocal space interesting properties of the aperiodic octagonal array can be provided through the experimental determination of the Fourier spectra of the samples. Diffraction patterns are produced as a result of the regional periodicity. Figure 5 shows the visible diffraction pattern produced by a normally incident green laser beam for the quasi crystal fabricated. The light source was a Ar^+ ^laser operating on the TEM_00 _mode at the wavelength λ = 514.5 nm and having a beam waist *w*_0 _= 2 mm. After having spatially filtered the laser beam through a lens-pinhole-lens system, the source is incident on the sample. Through the spatial filter, the incident light is diffracted creating an airy pattern: the central zero-order has an excellent spatial coherence and represents a good approximation of a plane wave. Higher diffraction orders have highly divergent wave vectors with respect to the optical axis. The second pinhole filters only the central spot of the airy pattern. An optical system consisting of two confocal lenses is placed between the two pinholes providing a magnification ratio of 3.47 that ensures a uniform illumination of the experimental sample having a square surface of ~700 μm^2^. A charge-coupled device (CCD) array coupled with a focusing lens system is finally used to acquire the Fourier spectra of the structure.

The diffraction pattern possesses eightfold rotational symmetry, and contains a series of spots of different intensity, surrounding the central undiffracted beam. These spots can be associated with vectors in reciprocal space. The observed peaks are sharp and symmetrically distributed. Each order has rings of spots at different cone angles around the zero-order central spot. The picture clearly reveals the presence of quasiperiodicity within the sample.

### Optical transmission measurements

The light propagating in the glass substrate which is extracted by diffraction was measured at room temperature using a multimode fiber, a CCD imaging telescope (OL610), and a CCD-based spectroradiometer (OL770-LED).

**Figure 6:**
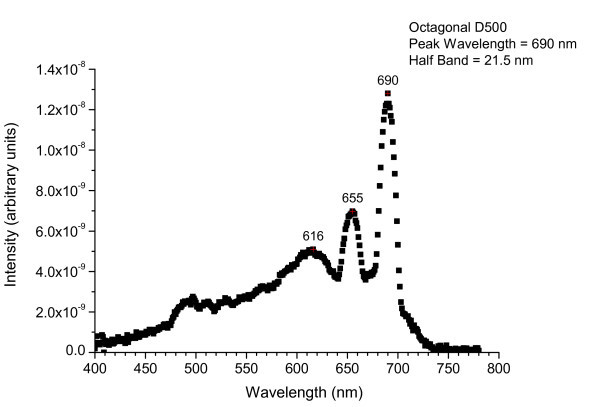
**Spectrally integrated light intensity characteristics of the realized hybrid nanostructure**. Measured emission profile of the fabricated sample as detected normally from the sample surface using a multimode fiber, a CCD imaging telescope, and a CCD-based spectroradiometer. A white light was introduced from an edge of the glass and was propagating inside the glass. The emission profile was measured along the ΓX direction of the octagonal QC. The main peak at 690 nm corresponds to the apparent resonant mode of the PC with a narrow FWHM of 21.5 nm.

A white light was introduced from an edge of the glass and was propagating inside the glass. The QPC area was seen as a red area because of the wavelength-selective diffraction by the hybrid QPC. Figure 6 shows spectrally integrated light intensity characteristics of the sample as detected normally from the sample surface. The sample shows three peaks (616, 655, and 690 nm) in the spectrum. The resonant peaks appear due to the QPC feedback effect, and the full width at half-maximum (FWHM) of spectrum is of 21.5 nm at the main peak. These resonant peaks are considered to originate from different transverse guiding modes with different modal indices, which are diffracted to the normal direction by the QPC effect.

It has been studied that when the period of a two-dimensional photonic crystal is equal to the cavity wavelength of the guided mode, the guided waves propagating to several in-plane directions are coupled to the radiation mode in the direction normal to the device surface since the Bragg diffraction condition is satisfied. The vertical extraction of the light, by the coupling of the modes guided by the PC slab to the free radiation via Bragg scattering, is ruled by a phasematching condition, namely by the conservation of the in-plane component of the momentum at the air-dielectric interface [43].

## Conclusions

In this work we patterned, for the first time to our knowledge, a nanocomposite containing colloidal semiconductor quantum rods of nanometer size scale to fabricate a novel 2D hybrid eightfold symmetric aperiodically ordered PQC. Our hybrid organic/inorganic nanocomposite is formed by using inorganic NRs, core/shell CdSe/CdS quantum rods, as inclusions in an organic polymer matrix (PMMA). Our nanocomposite has been prepared as a film in which each domain/inclusion can perform a specific photonic or optoelectronic (combined electronic and photonic) function. This permits introducing multifunctionality, and each of these functions can independently be optimized.

A semiconductor nanocrystals based 2D QPC pattern with rods with a diameter of 500 nm and a depth of 700 nm has been uniformly formed by EBL in a large area of 650 × 650 μm^2^. The diffraction effect of our sample has been confirmed and consists of a narrow red emission with a FWHM of 21.5 nm. The measured resonances peaks are due to the QPC feedback effect.

The possibility to pattern hybrid materials open the route to the development of new high performing optical devices such as organic light-emitting diodes, ultra-low threshold lasers, and non-linear devices. In a recent work of some of the authors [36] CdSe/CdS colloidal quantum rods have already proved to be very suitable in lasing applications. In future studies we shall focus our attention to lasing effects obtained in hybrid QPC microcavities and based on band edge engineering.

## Abbreviations

CCD: charge-coupled device; EBL: electron beam lithography; FDTD: finite difference time domain; FWHM: full width at half-maximum; IPA: isopropanol; ITO, indium tin oxide; MIBK, methyl isobutyl ketone; NRs: nanorods; 1D: one-dimensional; PQCs: photonic quasicrystals; PMMA: polymethylmethacrylate; PBGs: photonic band-gaps; SEM: scanning electron microscopy; TEM: transmission electron microscopy; TEM: transverse electromagnetic; 2D: two-dimensional.

## Competing interests

The authors declare that they have no competing interests.

## Authors' contributions

LP conceived of the study, carried out the nanocomposite preparation and the fabrication of the samples, the SEM characterizations and drafted the manuscript. MR carried out the far field diffraction characterization and the spectra measurements. LM supervised the synthesis of colloidal nanorods and provided the TEM characterization. MZ prepared NR solutions. JZ participated in the design of the study. PM participated in the design of the study and its coordination and helped to draft the manuscript. All authors read and approved the final manuscript.
